# Improving radiomic model reliability using robust features from perturbations for head-and-neck carcinoma

**DOI:** 10.3389/fonc.2022.974467

**Published:** 2022-10-14

**Authors:** Xinzhi Teng, Jiang Zhang, Zongrui Ma, Yuanpeng Zhang, Saikit Lam, Wen Li, Haonan Xiao, Tian Li, Bing Li, Ta Zhou, Ge Ren, Francis Kar-ho Lee, Kwok-hung Au, Victor Ho-fun Lee, Amy Tien Yee Chang, Jing Cai

**Affiliations:** ^1^ Department of Health Technology and Informatics, The Hong Kong Polytechnic University, Hong Kong, Hong Kong SAR, China; ^2^ Department of Clinical Oncology, Queen Elizabeth Hospital, Hong Kong, Hong Kong SAR, China; ^3^ Department of Clinical Oncology, The University of Hong Kong, Hong Kong, Hong Kong SAR, China; ^4^ Comprehensive Oncology Centre, Hong Kong Sanatorium and Hospital, Hong Kong, Hong Kong SAR, China

**Keywords:** radiomics, head and neck squamous cell carcinoma, model reliability, feature reliability, model robustness

## Abstract

**Background:**

Using high robust radiomic features in modeling is recommended, yet its impact on radiomic model is unclear. This study evaluated the radiomic model’s robustness and generalizability after screening out low-robust features before radiomic modeling. The results were validated with four datasets and two clinically relevant tasks.

**Materials and methods:**

A total of 1,419 head-and-neck cancer patients’ computed tomography images, gross tumor volume segmentation, and clinically relevant outcomes (distant metastasis and local-regional recurrence) were collected from four publicly available datasets. The perturbation method was implemented to simulate images, and the radiomic feature robustness was quantified using intra-class correlation of coefficient (ICC). Three radiomic models were built using all features (ICC > 0), good-robust features (ICC > 0.75), and excellent-robust features (ICC > 0.95), respectively. A filter-based feature selection and Ridge classification method were used to construct the radiomic models. Model performance was assessed with both robustness and generalizability. The robustness of the model was evaluated by the ICC, and the generalizability of the model was quantified by the train-test difference of Area Under the Receiver Operating Characteristic Curve (AUC).

**Results:**

The average model robustness ICC improved significantly from 0.65 to 0.78 (P< 0.0001) using good-robust features and to 0.91 (P< 0.0001) using excellent-robust features. Model generalizability also showed a substantial increase, as a closer gap between training and testing AUC was observed where the mean train-test AUC difference was reduced from 0.21 to 0.18 (P< 0.001) in good-robust features and to 0.12 (P< 0.0001) in excellent-robust features. Furthermore, good-robust features yielded the best average AUC in the unseen datasets of 0.58 (P< 0.001) over four datasets and clinical outcomes.

**Conclusions:**

Including robust only features in radiomic modeling significantly improves model robustness and generalizability in unseen datasets. Yet, the robustness of radiomic model has to be verified despite building with robust radiomic features, and tightly restricted feature robustness may prevent the optimal model performance in the unseen dataset as it may lower the discrimination power of the model.

## Introduction

Radiomics is an emerging artificial intelligence technology that utilizes high-throughput features extracted from imaging features for divulging cancer biological and genetic characteristics (1–4) in oncology. It has demonstrated promises and offered insights with its defined radiomic models into cancer diagnosis (5), prognostication (6), treatment response (7) as well as toxicity prediction (8). Despite a wide range of potential applications in the clinic, a primary concern of radiomics modeling is its robustness of radiomic models.

Identifying robust features is the prerequisite for building a robust radiomic model. However, the rare availability of test-retest scans prevents radiomic studies from comprehensively assessing feature robustness. Therefore, Zwanenburg et al. (9) proposed a perturbation-based dataset-specific radiomic feature robustness assessment method, an alternative to the conventional test-retest method. The feature robustness is quantified using the intra-class coefficient of correlation (ICC) from simulated perturbation images. The quantified feature robustness is used to identify and remove the low-robust features. However, the impact of eliminating low-robust features in radiomic modeling on the final model has not been discussed, which prevents the optimal utility of feature robustness.

Therefore, it would be instructive if the impact on radiomic model is clear when removing low-robust features. This manuscript evaluated the radiomic model’s robustness and generalizability under different thresholds of the low-robust feature removal. The model robustness is quantified with ICC using the perturbation method (10), and model generalizability is quantified with the train-test difference of Area under the Receiver Operating Characteristic curve (AUC), AUC_testing_ – AUC_training_. The change in the model performance would provide informative guidance when removing low-robust radiomic features from modeling.

## Materials and methods

### Overview

**Figure 1:**
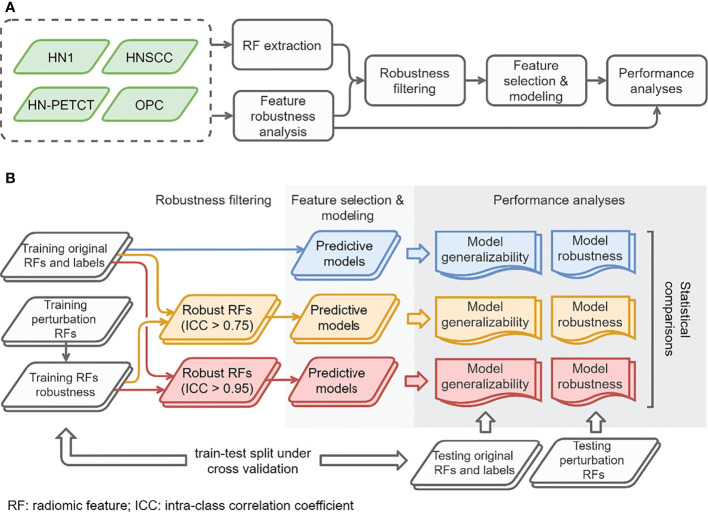
The overall study workflow **(A)** and model construction and performance analyses workflow **(B)**.

The overall study workflow is summarized in **Figure 1A**. Four publicly available datasets of head-and-neck cancer (HNC) named 1) Head-Neck-Radiomics-HN1 (HN1) (1, 11),, 2) Head-Neck-PET-CT (HN-PETCT) (11, 12),, 3) HNSCC (13–15), 4) OPC-Radiomics (OPC) (16, 17), were collected, and each dataset was used to perform the analysis independently. Two prediction outcomes, including distant metastasis (DM) and local-/regional- recurrence (LR), were modeled using five commonly used classifiers. The five classifiers include Ridge (18), Supporting Vector Classifier (SVC) (19), classifiers implementing the k-nearest neighbor’s vote (KNN) (20), Decision Tree (21), and Multilayer Perceptron Neural Network (MLP) (22). Each dataset was randomly split into multiple training and testing cohorts for repeated stratified cross-validation, and the training cohorts underwent robustness analysis, feature selection, and modeling. During each cross-validation iteration, the robustness of each radiomic feature was analyzed by image perturbations on the training samples and quantified by ICC. Features with high robustness scores were filtered out and further selected based on outcome relevance and redundancy before model training. To validate the performance of both model generalizability and robustness using radiomic features with increasing robustness, three groups of radiomic models were constructed 1) without feature robustness filtering, 2) with filtering threshold of 0.75, and 3) with filtering threshold of 0.95, as shown in **Figure 1B**. The robustness and generalizability of the three groups of radiomic models were compared statistically. The comparisons were performed independently for the 4 datasets, 2 outcomes, and 5 classifiers, resulting in 40 experiments in total. The improvements of the final selected radiomic feature robustness were also validated through statistical comparisons.

### Materials

A total of 1,419 HNC patients were recruited from the four publicly available datasets from The Cancer Imaging Archive (TCIA) (20). Pre-treatment computed tomography (CT) images and corresponding structure sets for radiation therapy were collected in DICOM format from the TCIA website. DM and LR records were also collected as predictive targets for radiomic modeling. They are two critical oncological endpoints in cancer treatment prognosis (23, 24), and the common predictive outcomes in many radiomics studies (6, 25, 26).

In order to ensure data consistency, a set of inclusion criteria were applied. Only patients with available 1) pre-treatment CT images, 2) clinical outcomes records of both DM and LR, and 3) primary gross tumor volume (GTV) contours were included in the study. The identifier of the selected image and the GTVs are also shared in GitHub for replication purposes. Each dataset was splitted into 60 training and testing sets using repeated stratified cross-validation. The folder numbers were chosen in a way that at least two patients in the minority group and 100 patients in total are left for testing to ensure the reliability of the testing performance. The final selected patient numbers, patient distributions for the two prediction outcomes, and train-test split cross-validation methods for the five datasets are listed in **Table 1**.

**Table 1:** The total patient numbers, patient distributions of the two binary prediction outcomes, and the train-test cross-validation methods of the screened patient cohort of the four public datasets.

Dataset name	Total patient No.	Distant metastasis		Local-/regional- recurrence		Cross-validation method
		Event	Non-event	Event	Non-event	
HN1	137	8	129	34	103	Stratified 2-fold, 30 repetitions
HN-PETCT	298	40	258	43	255	Stratified 3-fold, 20 repetitions
HNSCC	460	39	421	65	395	Stratified 4-fold, 15 repetitions
OPC	524	74	450	73	451	Stratified 4-fold, 15 reptations

### Image preprocessing and radiomic feature extraction

Radiomic features were extracted from the pre-treatment CTs within GTVs. The images and GTV contours were preprocessed before extracting features to maintain feature reproducibility and consistency (27, 28). First, CT images were isotopically resampled into 1mm x 1mm x 1mm using B-spline interpolation. The GTV contours were converted into voxel-based segmentation masks according to the resampled CT image grids. Additionally, a re-segmentation mask of the HU range of [-150, 180] was generated for each image to limit the texture feature extraction within soft tissue. All the mentioned preprocessing steps were implemented on Python (3.8) using SimpleITK (1.2.4) (29) and OpenCV (30) packages.

The rest of image preprocessing and radiomic feature extraction were performed using Pyradiomics (2.2.0) (31) package. In addition to the original image, features were extracted from 11 filtered images, including three Laplacian-of-Gaussian (LoG) filtered images (with a sigma value of 1, 3, and 6 mm), and eight coilf1 wavelet filtered images (LLL, HLL, LHL, LLH, LHH, HLH, HHL, HHH). The image intensities of both the original and filtered images were discretized into multiple fixed bin counts of 50, 100, 150, 200, 250, 300, and 350 for texture feature extraction to reduce the feature susceptibility to image noise. A total of 5486 radiomics features were extracted for each patient. The radiomic feature extraction parameter file for Pyradiomics can be found in the GitHub link.

### Feature robustness analysis and filtering

The robustness of radiomic features were analyzed *via* the image perturbations in four modes proposed by Zwanenburg et al. (9) with slight modifications. For each perturbation, both the image and mask were translated and rotated simultaneously by a random amount. They aim to simulate the patient position variation during imaging. A random Gaussian noise field was added to the image to mimic the noise level variations between different imaging acquisitions. The GTV mask was also deformed by a randomly generated deformable vector field. It aims to mimic the inter-observer variability during GTV delineation. Dice similarity index of 0.75 and the Hausdorff distance of 5 mm were used to constrain the perturbed contours. Multiple parameters of the different perturbation modes were chosen. The translation distances, rotation angles, noise addition levels, and contour randomization parameters were listed in **Table 2**. To explore the perturbations within the specified range as much as possible, 60 perturbations among the entire 4,423,680 combinations of perturbation modes were randomly chosen independently for each patient. The complete set of radiomic features were extracted for the chosen perturbations, and the feature robustness was calculated for each training set using the one-way, random intraclass coefficient of correlation (ICC) (32, 33), with patients as subjects and perturbations as raters. The ICC scores were used to filter out the robust features based on a pre-defined threshold before feature selection and modeling

**Table 2:** The parameters of perturbation modes.

Perturbation modes	Perturbation range	Reference axis	Perturbation number	Total number
Translation distance (mm)	0 to 3 with a 0.2 step size	AP, SI, LM	4,096	4,423,680
Rotation angles (degree)	-20 to 20 with a 5 step size	SI	9	
Noise addition level	0, 1, 2, 3	–	4	
Contour Randomization	30	–	30	

AP, anterior-posterior; SI, superior-inferior; LM, lateral-medial.

### Feature selection and modeling

A two-step feature selection approach was adopted to obtain the top features that are less redundant and more relevant to the outcome for modeling. First, the outcome relevance of each feature was evaluated by one-way ANOVA P-value repeatedly under downsample bootstrapping [imbalanced-learn 0.8.0 (34)] without replacement with 100 iterations on the training set. Features with P-values less than 0.1 were picked out in each iteration and ranked by their frequencies, and the top 10% features with the highest frequencies were chosen. Second, the feature with a higher mean correlation with the rest of the features in each highly correlated feature pair was removed. Pearson correlation coefficient was used to evaluate inter-feature correlation, and the threshold of 0.6 was chosen to identify the feature pairs with high correlations. A maximum of 10 features was further filtered based on the outcome relevance frequency ranking acquired in the previous step. The predictive models were trained from the final selected features using five different classification methods with automatic hyperparameter tunning. All the model trainings were implemented with the scikit-learn (0.24.0) (35) package. All the feature selection and modeling process was on training dataset.

### Performance analyses

The reliability of the predictive models was evaluated in both generalizability and robustness. Model generalizability evaluates model predictability consistency between the training cohort and the unseen cohort. It is quantified as the difference between training and testing predictability which is scored by the AUC. The model robustness metric was designed to evaluate the prediction reliability of patients under different perturbations across all the patients using ICC (1,1) (10). The ICC(1,1) is calculated with


$$\frac{MS_{R}-MS_{W}}{MS_{R}+\left(k+1\right)MS_{W}}$$

where MS_R_ = mean square for rows; MS_W_ = mean square for residual sources of variance; k = number of raters/measurements. In our case, MS_R_ is the mean square for patients, and MS_W_ is the averaged inter-perturbation variance, and k is the number of perturbations.

These two performance scores were calculated for all the models generated from the 60 cross-validation iterations and statistically compared between each of the two feature robustness filtering thresholds (ICC > 0.75, ICC > 0.95) and the performance of models constructed without robustness filtering using pairwise t-test. The comparisons were performed for each dataset, prediction outcome, and modeling classifier independently. Additionally, the robustness of the final selected features with and without robustness filtering was statistically compared by pairwise t-test for each dataset and prediction outcome.

### Bias evaluation against feature selection method

It is possible that the single feature selection method could lead to bias in the results. To facilitate the potential bias, the minimum redundancy maximum relevance (mRMR) feature selection method was implemented. The robustness and generalizability analysis was performed for all four datasets and outcomes with the Ridge classifier. This bias evaluation aims to ensure that the conclusion is not biased towards a specific feature selection method.

## Results

### Feature robustness and model robustness

**Figure 2:**
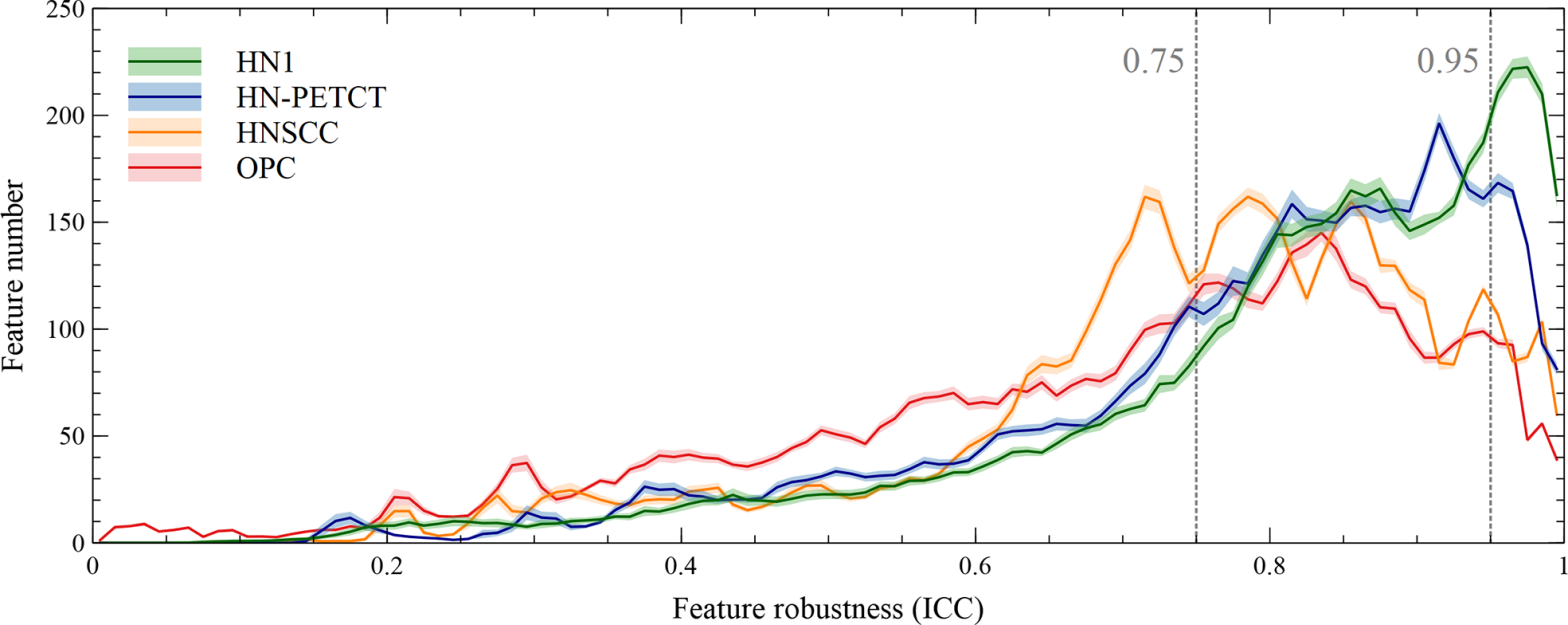
Histograms of the robustness of all the extracted radiomic features for the four analyzed datasets averaged under cross-validations. Feature robustness is quantified as intraclass correlation coefficient (ICC). The shaded areas indicate the 95% confidence interval of the average histogram curves. In general, there are more high-robust features than ones with low robustness. Different datasets show distinctive patterns of feature robustness distributions. HN1 and HN-PETCT have more features with high robustness, whereas HNSCC and OPC have the histograms skewed towards the lower end.

**Figure 3:**
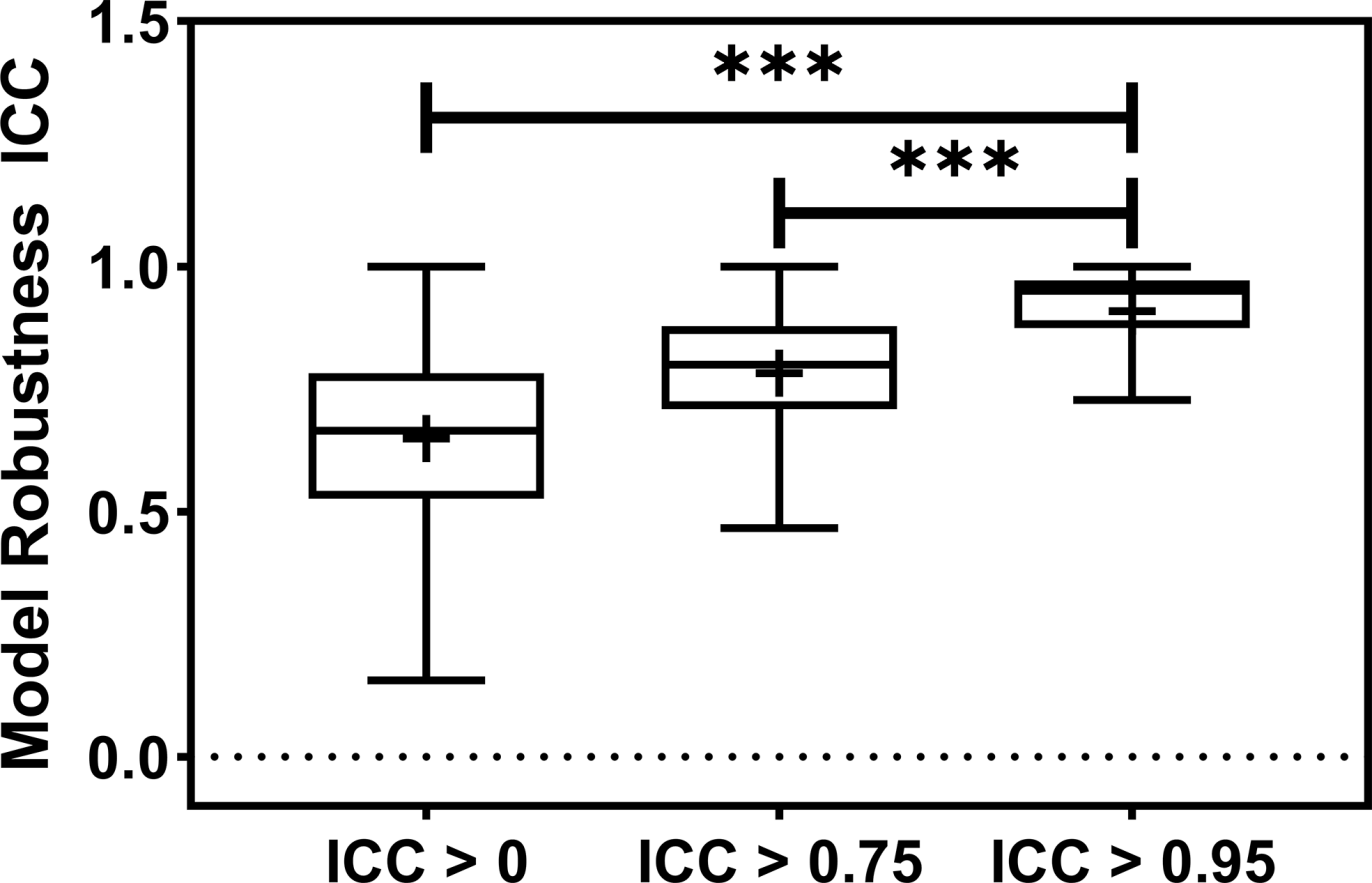
The barplot shows the model robustness ICC distribution for three feature robustness filtering groups, ICC > 0, ICC > 0.75, and ICC > 0.95. The feature robustness filtering of ICC > 0.95 yields the most robust model. *** indicates the p-value is smaller than 0.0001.

The radiomic feature robustness was quantified by the ICC under image perturbations. The distributions of all the extracted radiomic features show a strong skewness towards higher robustness, as shown by the histograms of feature ICCs for the four datasets in **Figure 2**. Different datasets show distinctive patterns of feature robustness distributions. HN1 (median = 0.84) and HN-PTECT (median = 0.82) has more features with high robustness whereas HNSCC (median = 0.77) and OPC (median = 0.74) have the histograms skewed towards the lower end. On average, 3320/5486 radiomic features remained after being filtered by the threshold of 0.75 and 605/5486 remained for the threshold of 0.95. The final selected radiomics features after the subsequent feature selection procedures showed a significant increase (P< 10^-11^) in mean ICC with increasing feature robustness filtering thresholds. On average, the ICC of the final selected features improved by 0.18 under the filtering threshold of 0.75, and the improvement increased to 0.30 under the threshold of 0.95, as shown by the first column of the heatmaps in **Figure 3A**.

The radiomic model robustness improved significantly after removing non-robust features prior to modeling. The ICC of radiomic models constructed without feature robustness filtering is 0.65 averaged over all the datasets, outcomes, and classifiers. It is improved to 0.78 (P< 0.0001) and 0.91 (P< 0.0001) after feature robustness filtering with ICC > 0.75 and ICC > 0.95, respectively. The box plot in **Figure 3** showed the distribution of the model robustness ICC. Interestingly, the outliers indicated observations in low robust models (ICC< 0.5) using high robust features (ICC > 0.75 and ICC > 0.95), despite the statistical significance in model robustness differences. The outlier samples were further analysed in terms of the datasets and classifiers. No statistical difference was found in different datasets (P > 0.05), and statistical differences were observed in the classifiers. In the feature robustness filtering group of ICC > 0.95, 8 (0.33%) samples in KNN, and 4 (0.17%) samples in Decision Tree were found to have poor model robustness performance despite using excellent-robust radiomic features.

**Figure 4:**
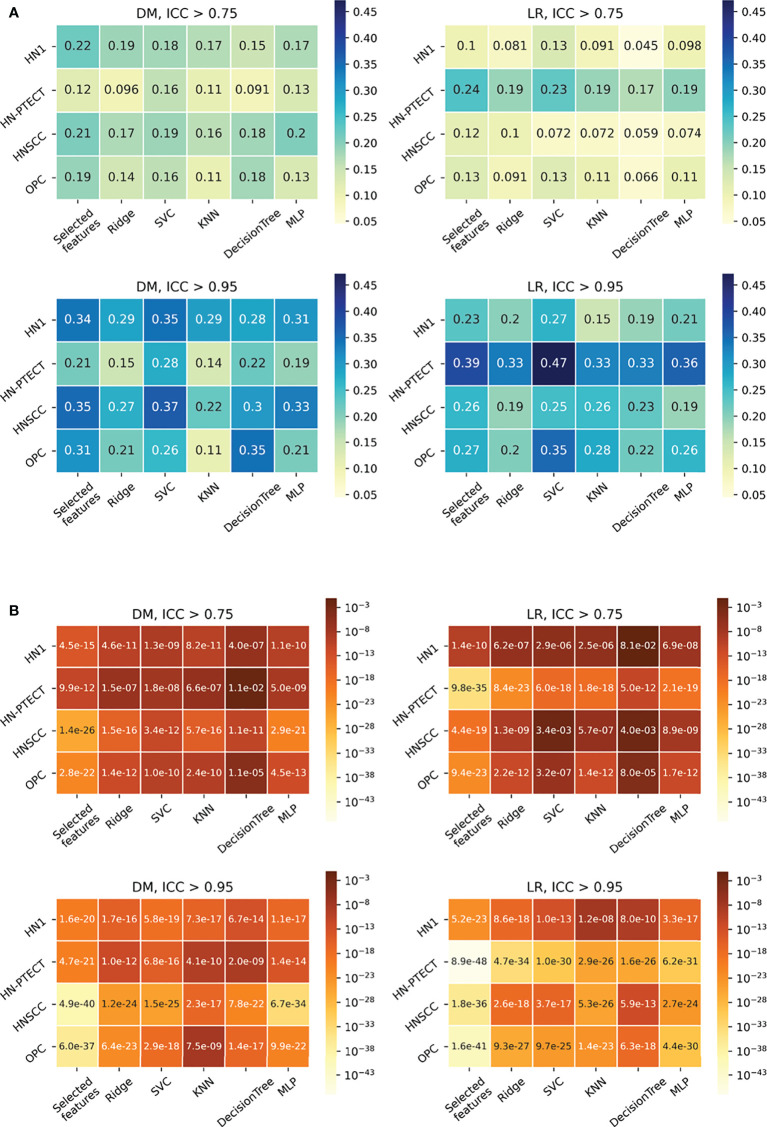
Average intraclass correlation coefficient (ICC) improvement **(A)** and t-test P-values **(B)** of the final selected features and testing predictions after robust feature pre-selection shown in heatmaps. Each heatmap contains the results of one prediction outcome and one feature robustness filtering threshold. The first column of each heatmap represents the improvements of the final selected radiomic features, and the remaining five columns are the improvements of the testing prediction robustness using different classifiers. Results of the four datasets are recorded in rows. All the experiments showed positive improvements in ICC. A higher and more statistically significant increase in average ICC improvements can be observed with a higher filtering threshold.

The detailed results in model robustness improvements and their statistical tests for the four datasets (row) and five classifiers (column) are visualized in the last five columns of the heatmaps in **Figure 4**, separated by outcome and robustness filtering thresholds. Heterogeneous model robustness improvements can be observed in different datasets, classifiers, and prediction outcomes. Higher (ICC > 0.75: 0.045~0.24, ICC > 0.95: 0.11~0.47) and more statistically significant (ICC > 0.75: P-value=9.8 × 10^-35^~1.1 × 10^-2^, ICC > 0.95: P-value=8.9 × 10^-48^~1.2 × 10^-8^) prediction ICC increases were found with the higher feature robustness filtering threshold in general.

### Model generalizability

**Figure 5:**
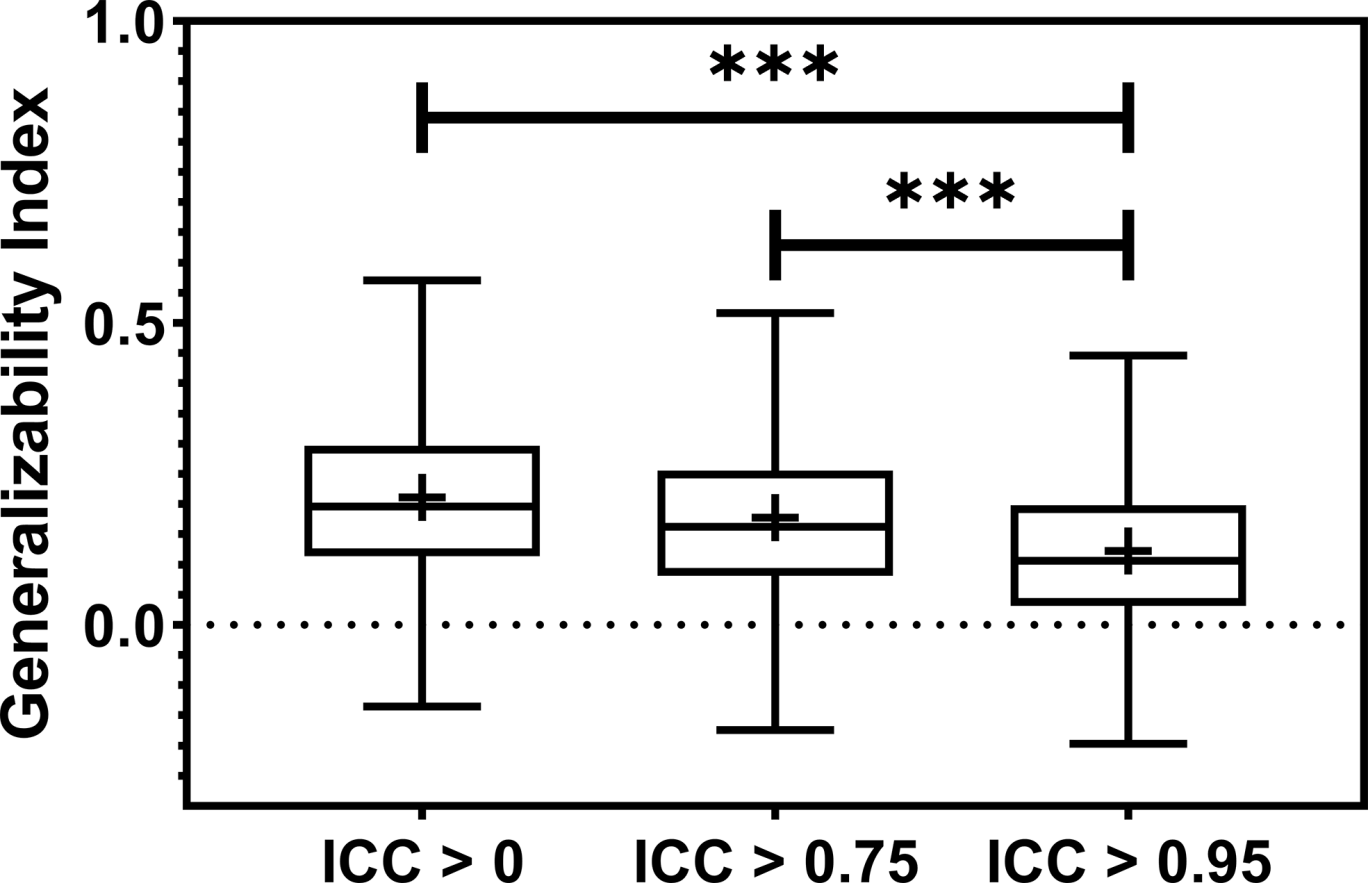
The boxplot showed the train-test performance differences. The most restricted feature robustness filtering provides the most generalizable models. *** indicates the p-value is smaller than 0.0001.

Model generalizability is quantified as the difference between the training and testing Area Under the Receiver Operating Characteristic Curve (AUCs), and a lower score indicates better generalizability. The model generalizability score averaged over all the datasets, outcomes, and classifiers are 0.21, 0.18, and 0.12 without robustness filtering, with the filtering threshold of 0.75 (P< 0.0001) and the threshold of 0.95 (P< 0.0001), respectively, shown in **Figure 5**.

**Figure 6:**
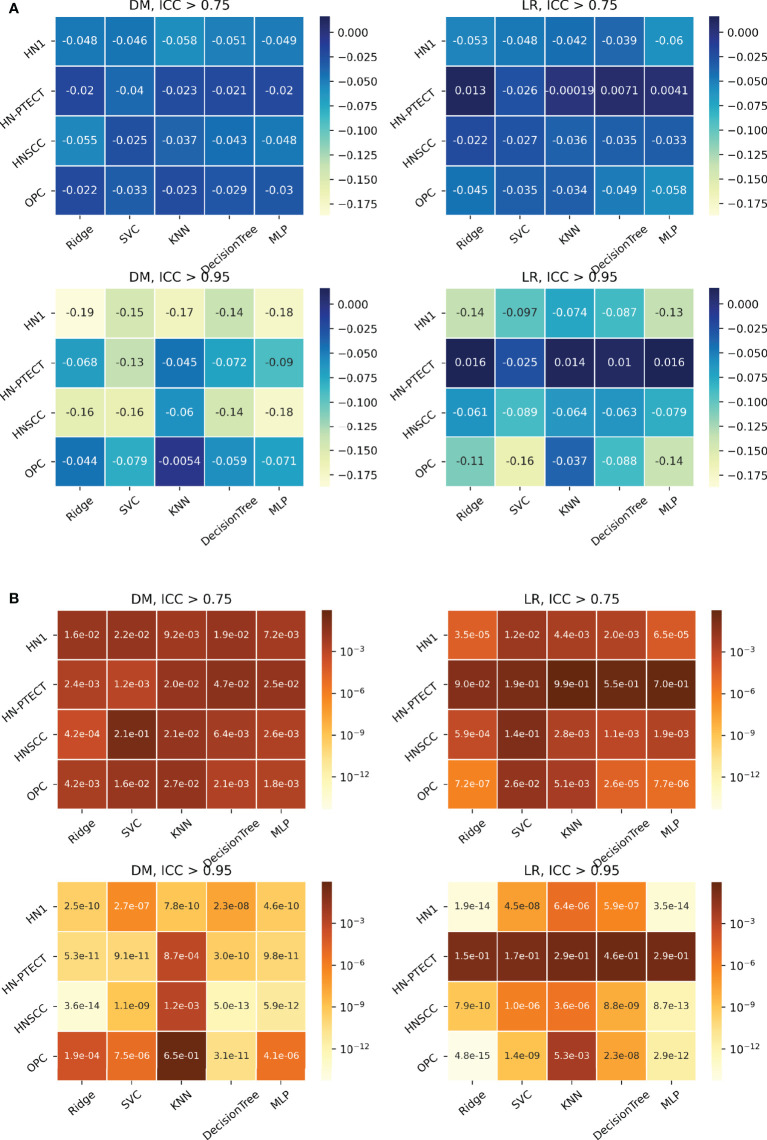
Heatmaps on mean model generalizability improvements **(A)** and statistical test results **(B)** after feature robustness filtering. Model generalizability is defined as the difference between training and testing AUCs, AUC_testing_ - AUC_training_. A score closer to zero shows better generalizability. In general, model generalizability improved after feature robustness filtering, as shown by the negative values on the heatmaps **(A)** for both filtering thresholds. Greater improvements were observed with the higher filtering threshold (ICC > 0.95). Moreover, more significant differences are shown by the smaller P-value. However, the predictions of LR on the dataset HN-PETCT showed worse generalizability after feature robustness filtering and the opposite trend of generalizability change and statistical test results with increasing filtering thresholds.

**Figure 7:**
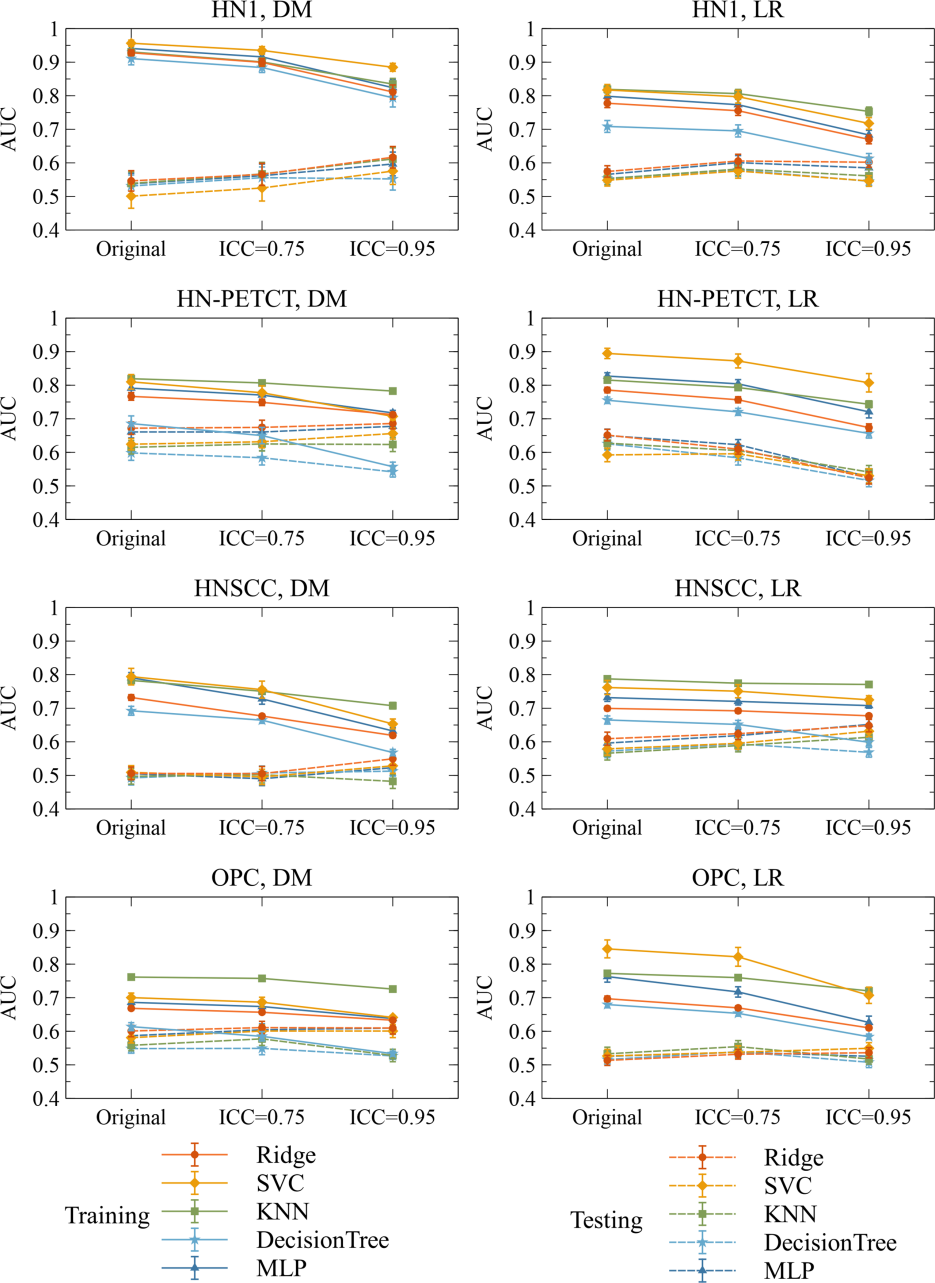
The mean and its 95% confidence interval of the training and testing AUCs of the final constructed models. Each color represents one classifier for modeling. The solid lines represent the training performances, and the dashed lines represent the testing performances. The 95% confidence intervals are drawn by the error bars. Each subfigure contains the evolution of training/testing AUCs with increasing feature robustness filtering thresholds for one dataset and prediction outcome. A decreasing trend of training AUCs were observed with increasing thresholds for all the datasets, prediction outcomes, and classifiers. The testing AUCs remain stable except for local-regional recurrence prediction on HN-PETCT dataset.

**Figure 6** shows the subgroup analysis based on datasets, outcomes and classifiers. In general, model generalizability showed statistically significant improvements after feature robustness filtering in most experiments, as shown by the majority of negative mean generalizability differences and small t-test P-values. However, the prediction of LR on HN-PETCT had positive mean generalizability differences (ICC > 0.75: -0.026~0.013, ICC > 0.95: -0.025~0.016) for most of the classifiers under both filtering thresholds. Despite the heterogeneous results among datasets, outcomes, and classifiers, larger improvements with higher statistical significance in mode generalizability were observed with the higher feature robustness filtering threshold (ICC > 0.75: -0.06~-0.02, P-value = 7.2 × 10^-7^~2.1 × 10^-1^; ICC > 0.95: -0.19~-0.054, P-value=4.8 × 10^-15^~6.5 × 10^-1^) apart from LR models for HN-PETCT. **Figure 7** shows the comparisons of average training and testing AUCs along with its 95% confident interval across the cross-validation models with increasing feature robustness filtering thresholds. Each subfigure contains the results of all the five classifiers shown in different colors and separated by datasets and clinic outcomes. Decreasing training AUCs were observed with increasing filtering thresholds. Specifically, the training AUCs averaged over all the datasets and prediction outcomes without feature robustness filtering, with robustness filtering on ICC > 0.75, and with filtering on ICC > 0.95 are 0.78, 0.76, and 0.69, respectively. Significant drops of training AUCs (pairwise t-test P-values< 0.05) were observed in 33/40 experiments from no feature robustness filtering to the threshold of 0.75 and 40/40 experiments to the threshold of 0.95. Meanwhile, the average testing AUCs are 0.57, 0.58, 0.57 with 18/40 experiments showing statistical significant difference (pairwise t-test P-values< 0.05) for ICC > 0.75 and 24/40 for ICC > 0.95. Different classifiers showed heterogeneous trends of testing AUCs under increasing thresholds. Notably, the testing AUCs of LR radiomic models on HN-PETCT showed significant decrease for feature robustness filtering with ICC > 0.75 (mean decrease: 0.026, 5/5 classifiers with P-value< 0.05) and ICC > 0.95 (mean decrease: 0.102, 4/5 classifiers with P-value< 0.05).

### Bias evaluation

The model robustness improved significantly with the improved feature robustness *via* the mRMR feature selection, as shown in **Table 3**, which is consistent with the model robustness improvement with filer-based feature selection.

**Table 3:** The model robustness (ICC) for different feature robustness pre-screening thresholds.

Outcomes		ICC > 0	ICC > 0.75	ICC > 0.95
DM	HN1	0.73 (0.66 - 0.79)	0.88 (0.84 - 0.91)	0.95 (0.94 - 0.96)
	HN-PETCT	0.76 (0.71 - 0.80)	0.92 (0.90 - 0.94)	0.92 (0.97 - 0.98)
	HNSCC	0.69 (0.64 - 0.75)	0.78 (0.93 - 0.82)	0.94 (0.93 - 0.96)
	OPC	0.74 (0.70 - 0.79)	0.91 (0.90 - 0.93)	0.99 (0.99 - 0.99)
LR	HN1	0.70 (0.64 - 0.77)	0.86 (0.82 - 0.90)	0.96 (0.95 - 0.98)
	HN-PETCT	0.63 (0.57 - 0.70)	0.81 (0.77 - 0.85)	0.94 (0.92 - 0.95)
	HNSCC	0.73 (0.68 - 0.78)	0.89 (0.86 - 0.91)	0.98 (0.97 - 0.98)
	OPC	0.70 (0.66 - 0.75)	0.84 (0.81 - 0.87)	0.97 (0.97 - 0.98)

The training AUC showed a consistent drop with the increase in the threshold of feature robustness, shown in **Table 4**. In contrast, the testing AUC showed an increase or maintaining the same level, resulting in the improved model generalizability.

**Table 4:** The training and testing AUC between different feature robustness pre-screening thresholds.

Outcomes		ICC > 0		ICC > 0.75		ICC > 0.95	
		Training AUC	Testing AUC	Training AUC	Testing AUC	Training AUC	Testing AUC
DM	HN1	0.96	0.52	0.92	0.53	0.82	0.60
	HN-PETCT	0.84	0.69	0.82	0.70	0.74	0.70
	HNSCC	0.76	0.53	0.68	0.50	0.63	0.53
	OPC	0.72	0.60	0.68	0.62	0.64	0.62
LR	HN1	0.86	0.57	0.82	0.60	0.70	0.60
	HN-PETCT	0.83	0.62	0.79	0.63	0.70	0.54
	HNSCC	0.74	0.62	0.72	0.64	0.68	0.65
	OPC	0.72	0.52	0.69	0.54	0.61	0.54

The bias analysis against the feature selection method showed consistent results between the filter-based and mRMR feature selection methods in improving model robustness and generalizability with robust radiomic features. Therefore, it is unlikely that different feature selection algorithms would affect the conclusion.

## Discussion

After removing low-robust features, the radiomic model’s robustness and generalizability have been improved, and the improvement is consistent across multiple datasets, different clinical outcomes, and classifiers. Our results also offer two practical implications. The radiomic model’s robustness needs to be evaluated despite using high-robust radiomic features in modeling. The restricted thresholding on feature robustness would prevent the optimal performance of the radiomic model to the unseen dataset.

Previous literature has discussed the positive impact of robust feature pre-selection on radiomic model generalizability and robustness. For instance, Haarburger et al. (36) envisioned that robust-only features are more likely to lead to a more reliable radiomic model. Vuong et al. (37) obtained a radiomic model with multi-institutional datasets, which performed equally well as a model on a standardized dataset by including pre-screening on the robust features. Our results confirmed their envision and findings with quantifiable measurements of model robustness and generalizability improvements, providing concrete evidence of increased model stability after feature robustness filtering.

**Figure 8:**
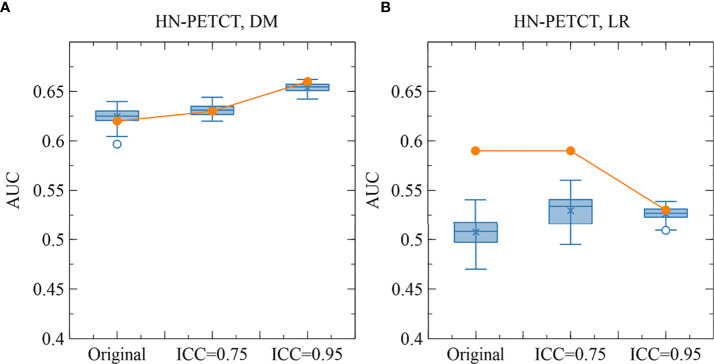
The comparison of the original and perturbed testing AUCs of HN-PETCT-298 averaged over train-test splits for the prediction of DM **(A)** and LR **(B)** using SVC. The testing AUCs showed high consistencies between the original images and perturbed images for the prediction of DM while large deviations were observed for the prediction of LR.

The improved model robustness can be explained by the reduced variability of the final selected features after pre-screening on feature robustness, as indicated by the statistically smaller mean feature ICCs. Model output variability is thus reduced as the final selected features are the direct model input. On the other hand, without feature robustness filtering beforehand, low-robust features are likely to remain after feature selection. They are more likely to be related to the outcome in the training cohort by chance (type I error) and less likely to be predictive of the unseen cohort or the entire population. Thus, the final constructed models tend to have high AUCs in training, but low testing. The high type I error caused by low feature robustness reduces the power of feature selection in identifying the truly predictive features and lowers the generalizability of the final constructed models. However, a statistically significant reduction (mean: 0.007, P-value< 0.001) in LR prediction generalizability and testing AUCs (mean: 0.1, P-value< 0.001) with pre-selection of robust features on the HN-PETCT dataset is discovered, as shown in **Figure 7**. We found out that one non-robust feature - *wavelet-LHH_glszm_ZoneEntropy* - demonstrated a significant correlation with LR in the entire HN-PETCT cohort with P-value< 0.001. Meanwhile, it is vulnerable against the image perturbations with an ICC of 0.36 (95% CI: [0.32, 0.42]) and thus removed from modeling, resulting in a reduction in overall model predictability and generalizability. This raises the concern about the limited reliability of testing predictability in representing the model generalizability on the unseen population. To further explain the reduced testing performance, we have calculated the distribution of testing AUCs on the perturbed data and compared with the results on the original data for dataset HN-PETCT and SVC classifier, as visualized in **Figure 8**. Compared with DM predictions, the testing AUCs for LR demonstrated higher variabilities, and the original testing AUCs deviated more to the averaged AUCs under perturbations. Although the original testing AUCs increased statistically (ICC< 0.75: mean increase = 0.02, P-value< 0.01; ICC< 0.95: mean increase = 0.019, P-value< 0.01) after feature robustness filtering for LR, the average testing AUCs showed the opposite trend. The high variability of testing AUCs on LR increases the risk of under-representative testing performance evaluation on the original data, which can be alleviated by feature robustness filtering. Our new findings also support recommendation of using the averaged feature values under image perturbations for modeling (9).

Notably, we applied a comprehensive evaluation framework to assess model robustness and generalizability under repeated cross-validations. Instead of only splitting the entire cohort into a single training-testing pair and generating a single model for evaluation, multiple independent train-test splits can give statistical and unbiased evaluations of the impact of radiomic feature robustness on model robustness and generalizability. The main drawback of this method is the high heterogeneity in training and testing performance among iterations (38), which may reduce the statistical significance of our results. We used image perturbations to assess both radiomic feature robustness and model robustness. Although the scope of the image perturbations applied in this study might be limited, and the resulting feature robustness and model robustness is not guaranteed to be as sensitive as test-retest imaging and manual re-contouring, they are rather conservative simulations that impose no additional cost in medical resources and can be easily applied to any dataset. Comprehensive validations of the proposed perturbation method in the future are warranted to increase the credibility of this work. There are other limitations of this study. First, we only considered four datasets of head-and-neck cancer datasets from The Cancer Imaging Archive (TCIA), and our results may only be generalizable to head-and-neck data. To further generalize the findings to other sites, it is encouraged to test our method on more cancer sites. Second, bias could arise from the single feature selection method, as different criteria and techniques in feature selection have different power in identifying truly predictive radiomic features. It is also suggested to validate our methods with different feature selection methods.

## Conclusion

In this study, we evaluated radiomic model’s robustness and generalizability by removing the low-robust features. Our results suggested to remove low-robust features to improve model robustness and generalizability to unseen data. Our findings also imply evaluating model robustness despite using robust features already, and the strictest threshold in feature robustness may undermine the optimal model performance.

## Data availability statement

The datasets presented in this study can be found in online repositories. The names of the repository/repositories and accession number(s) can be found below: https://github.com/vivixinzhi/improved-robustness-and-generalizability-of-radiomic-modeling-via-image-perturabtion.

## Author contributions

XT, JZ, and JC conceptualized the idea. XT, YZ, and JZ performed data analysis and validation. FK-hL, K-hA, VL, and AC provided the resources. ZM, SL, WL, and HX performed data cleaning and verification. TL, BL, TZ, GR, SL, WL, and HX provide the paper edition and review. XT drafted the original manuscript. All authors contributed to the article and approved the submitted version.

## Funding

This research was partly supported by Project of Strategic Importance Fund (P0035421), and Project of RI-IWEAR fund (P0038684) from Hong Kong Polytechnic University, and Shenzhen-Hong Kong-Macau S&T Program (Category C) (SGDX20201103095002019) from Shenzhen Science and Technology Innovation Committee.

## Conflict of interest

The authors declare that the research was conducted in the absence of any commercial or financial relationships that could be construed as a potential conflict of interest.

## Publisher’s note

All claims expressed in this article are solely those of the authors and do not necessarily represent those of their affiliated organizations, or those of the publisher, the editors and the reviewers. Any product that may be evaluated in this article, or claim that may be made by its manufacturer, is not guaranteed or endorsed by the publisher.
